# Minutes of the Third Annual Meeting of the Ohio Dental College Association

**Published:** 1855-04

**Authors:** Charles Bonsall

**Affiliations:** Sec’y.


### MINUTES OF THE THIRD ANNUAL MEETING OF THE OHIO DENTAL COLLEGE ASSOCIATION.

The Association met according to adjournment at 2 P. M., of Monday, Feb. 19, 1855, in the New Ohio Dental College buildings.

Present, Drs. Taylor, Taft, H. R. Smith, Duncan, J. M. Brown and Bonsall.

The minutes of last year were first read, and then the building committee handed in the different propositions they had received for the building, and gave some account of their labors in erecting the College building, and the further report was then postponed till 2 P. M., to-morrow.

Dr. C. B. Chapman was then appointed, as one of the medical examiners for to-morrow, in place of Professor L. M. Lawson, absent from the city, also Drs. J. S. King, and Watt, in place of Dr. Ulrey and Hamill, Dental Examiners, also absent.

Adjourned till 2 o'clock, P. M., to-morrow.

SECOND DAY.

Met according to adjournment, at 2 P. M.

Present, Drs. Taylor, Taft, A. S. Talbert, H. R. Smith, Bonsall, G. L. Payne, W. H. Goddard and J. P. Ulrey.

The report of the building committee in full was handed in, as follows:

*To the Ohio Dental College Association:*

The Committee to whom was entrusted the erection of the Ohio College of Dental Surgery, would respectfully report,

That the plan and proposals which were before the Association at our last meeting, were necessarily laid over for a few weeks, to enable the Committee to ascertain the additional cost of a stone front as then suggested, and by the time this was done, building materials and labor had so advanced in price, that the gentlemen whose proposals were then before us withdrew the same.

The Committee to secure every thing in the best manner and preclude the possibility of a long list of extras, determined to have a plan with regular and full specifications made by which the contract could be closed and made binding.

With this plan and specifications they gave out bids for the building, and received quite a number from different individuals, which we here present. The committee selected the lowest and most reliable one, and made a contract for the present building as it now stands. Amount of contract, $5400. They then employed Mr. Edwards to superintend the erection at $100; the vault for dissecting room proved defective and they had a new one made, for which they paid $65; they also paid for copper pipe running from dissecting room to vault, $80,75.

The account current, embracing all the moneys expended with the amount of cash received from sale of stock, initiation fees, the amount of money borrowed, &c., is herewith presented.

The Committee would remark, that the $500, which was to have been furnished by Mr. J. L. Talbott, and appropriated to the erection of the building, could not be realized. This rendered it necessary for the Committee to raise this additional amount of cash, to meet liabilities assumed, with the understanding that this would be furnished by him, through the Academy of Natural Sciences, in consideration of the use of the front room for ten years. They would also remark, that the dissecting room which they expected to have rented for a number of years for the sum of $250, was also not taken. These circumstances occurring at a time when the money market has been unusually depressed, has given them much difficulty in the raising of the amount of means promised in the contract.

These difficulties, however, were all met and have been overcome, and the Committee now have the use of all the means required to meet their engagements up to the first of April, when the amount due Messrs. Taylor and Talbott of $3000, will have to be paid, also, $1,723 83, which they are in debt for borrowed money.

They have the following arrangement made for the liquidation of the debt, $4000, promised at that time on a mortgage of five years, at ten per cent and the taxes.

The following amount is due on stock $200, and the following members of the Profession, have each subscribed a share of stock, which will be paid into the Treasury in the course of a year—Drs. T. L. Waring, Zanesville, O.; G. J. Friedricks, New Orleans and H. J. B. McKellops, St. Louis, Mo.

The Committee think that the amount of debt which the $4000 received on mortgage does not liquidate, can be paid off within the year, by the amounts now due on stock, and that which can be raised by the sale of stock in that time, an d that the amount had better be borrowed until this is paid, so that the mortgage shall be for the least possible amount.

The amount of rent received on the building will more than meet the interest on the mortgage. This with the initiation fees and annual dues paid into the Association's Treasury, will form a sinking fund for the payment of the mortgage. But this will not be sufficient of itself, and hence the Committee would suggest the propriety of issuing an additional amount of stock, to meet this by the time it becomes due. Yet we would also suggest the propriety of limiting the amount to be issued, believing that at the most, not over thirty shares need be thus issued. This additional amount of stock it will be observed, does not in the least affect injuriously the interest of the present stock-holders. The committee herewith present their accounts, and would be glad to have them examined.

Respectfully, &c.,
JAMES TAYLOR,
CHARLES BONSALL.

The Committee are sorry that Dr. A. Berry who has been an efficient member is not with us, and permitted to unite with us personally in this report.

On motion the report was adopted, and Drs. A. S. Talbert, Payne and Taft, were appointed as an auditing committee, who after examining the accounts and vouchers, reported as follows.

"We the Committee, appointed to audit the account of the building Committee, having examined their accounts and vouchers, find that they have been correctly kept, and show an indebtedness of the Association of $1,723 83, in addition to the $3,000 due to the contractor on the 1st of April proximo."

A. S. TALBERT,
G. L. PAINE,
J. TAFT.

Dr. E. Collins of Connersville, Ind., a graduate of this school, being present, was proposed as a member, and having been unanimously elected, signed the constitution and paid the initiation fee of six dollars; also received six dollars annual dues from Dr. Samuel Griffiths, of Louisville, Ky.

On motion, a vote of thanks was tendered through Dr. J. M. Brown, to Julius B. Thompson, for one of his "*Blow Pipes*," presented to the College.

*The Faculty of the Ohio College of Dental Surgery would present the following brief Report:*

They commenced their regular course of lectures on the first Monday of November last, and have continued them up to the close of last week, with the exception of the usual vacation between Christmas and New Year.

They felt it a duty to make the requisitions such, that every student entering the class should be on an equal footing, hence they have exacted cash or as near as possible its equivalent for their tickets, trusting no one unless their references or themselves were known to be responsible. This with the hard times which began to be severely felt at the commencement of the session, has very much diminished the number of the class, from that which we had every reason to believe it would have been, and which we feel assured it will be another year.

They have also felt it their duty to so enforce the rules in regard to the requisitions for graduation, that the number presented for graduation is smaller than was expected. We feel assured, however, that these things must have a beneficial influence in advancing the cause of Dental Science. To further this special object, they also would recommend, that a Professorship of Chemistry be created, and a suitable man selected to fill that place. In making this recommendation, they do not design that the price of tickets should be increased accordingly, but feel willing that the gross amount should stand as at present. Thus diminishing the price of their several tickets.

The number of the class this session, they report at 17, and the graduating class, 5.

The graduating class have all attended two courses of lectures in this Institution, but one, and he had attended one full course of lectures in a regular medical school. The students matriculated are all required to take the whole of the tickets, and the graduating class are required to *attend* a full course in the Institution immediately preceding their graduation. We refer to these things, so that the Association may know the course we are pursuing, and that which we intend by their approbation to continue.

Which was accepted, and on motion of Dr. Bonsall, it was, *Resolved*, That the Board of Trustees be requested to create another Professorship in the College—being a chair of chemistry and metallurgy.

The Association now went into an election for officers to serve the ensuing year, when the following were found to have been elected, viz.

*President.*—DR. JAMES TAYLOR,

1*st Vice President.*—DR. JONATHAN TAFT.

2*d do do* DR. H. R. SMITH.

*Secretary.*—CHARLES BONSALL.

*Treasurer.*—DR. J. B. SMITH.

*Dental Examiners.*—Drs. J. P. Ulrey, of Rising Sun, Ind., T. R. Willard, of Dayton, O., and George W. Keely of Ox ford, Ohio.

*Medical Examiners.*—Drs. Wm. H. Mussey and O. M. Langdon.

On motion, adjourned till 2 P. M. to-morrow.

THIRD DAY.

Met at 2 P. M., according to adjournment.

The minutes of the previous meetings were now read and adopted, and on motion of Dr. Talbert, it was unanimously,

*Resolved*, That the Trustees be authorized to issue to each member of the present Faculty, one share of stock in payment for the furnace, chairs, tables, repairing back yard, painting of anatomical and dissecting room floor, lettering doors, lead pipes, hydrant, &c., which we find amounts in the gross to about $500 over and above the regular incidental expenses they are obliged to pay.

On motion of Dr. Ulrey, it was *Resolved*, That the Trustees be authorized to issue a sufficient number of shares of stock, to pay the indebtedness of the institution, when upon consultation, *thirty* additional shares were fixed on as the necessary number.

On motion of Dr. Goddard, it was unanimously *Resolved*, That the thanks of this Association be tendered to the building committee, who have so promptly and efficiently executed the work committed to their charge.

Upon going into an election for a suitable person, to recommend to the Board of Trustees to fill the proposed chair of chemistry and metallurgy. Dr. George Watt, of Xenia, Ohio, was unanimously recommended; and on motion of Dr. H. R. Smith, it was *Resolved*, That in case of the resignation of Dr. Thomas Wood, (who may be under the necessity of resigning before another term shall commence,) the faculty be authorized to nominate a candidate to the trustees to fill the vacancy.

On motion of Dr. Collins, it was *Resolved*, That we recommend to the Trustees, that the title of the chair now filled by Dr. James Taylor, of Practical Dentistry and Pharmacy, or as it now stands, Principles and Practice of Dental Surgery, be changed to that of Institutes of Dental Science.

Dr. Wm. H. Goddard, requested the privilege of changing his annual payment into a subscription for a share of stock, which was granted.

Also, Dr. Watt having subscribed for a share to be paid for in chemical apparatus, he was unanimously elected a member, as also the following gentlemen who each subscribed for stock, viz.,

Dr. W. R. Webster, Richmond, Ind.,
D. A. J. Reeve, Mt. Vernon, Ohio.
Dr. T. L. Waring, Zanesville, O.,
Dr. Charles E. Keels, New Orleans, La.,
Dr. George J. Friedricks, do do do
Dr. H. J. B. McKellops, St. Louis, Mo.

The minutes were now read and adopted, and a copy directed to be furnished by the Secretary to the Dental Register for publication.

Adjourned to meet in the College building, on the third Monday of February next at 2 P. M.

CHARLES BONSALL, *Sec'y.*

